# Strain-encoded imaging for prediction of functional recovery in patients after acute myocardial infarction

**DOI:** 10.1186/1532-429X-11-S1-O31

**Published:** 2009-01-28

**Authors:** Mirja Neizel, Grigorios Korosoglou, Tim Schaeufele, Dirk Lossnitzer, Harald P Kuehl, Evangelos Giannitsis, Malte Kelm, Hugo A Katus, Nael F Osman, Henning Steen

**Affiliations:** 1 University Hospital Aachen, Aachen, Germany; 2 University Hospital Heidelberg, Heidelberg, Germany; 3 Johns Hopkins University, Baltimore, MD USA

**Keywords:** Acute Myocardial Infarction, Acute Myocardial Infarction, Functional Recovery, Steady State Free Precession, Microvascular Obstruction

## Introduction

Evaluation of reversible dysfunction after acute myocardial infarction (AMI) has important therapeutic and prognostic implications. The role of impaired systolic function for evaluation of functional recovery has been extensively investigated. However, whether impaired regional diastolic function after AMI also has predictive implications has not been investigated so far in humans using magnetic resonance imaging (MRI). Recently, Strain-Encoded (SENC) Imaging was introduced as a new MR-technique to evaluate myocardial strain and strain rate. SENC, compared to MR tagging, is a method that does not suffer that much from diastolic fading. Therefore, SENC is an ideal MR-method to determine not only regional systolic but also regional diastolic function.

## Purpose

To evaluate the predictive value of regional systolic and diastolic function for improvement of regional myocardial function in patients after AMI.

## Methods

MRI (1.5 T, Achieva, Philips, the Netherlands) was performed to 23 consecutive patients (mean age 57 ± 10) 3 ± 1 days after successfully reperfused ST-elevation-myocardial infarction and at a follow-up of 6 ± 2 month. 10 age-matched volunteers served as controls. True cine sequences of 3 long-axis views (2-,3- and 4-chamber) and a short-axis (SA) view covering the ventricle from apex to basis were acquired using a Steady State Free Precession (SSFP) sequence. After that, SENC cine images were acquired on the same long-axis planes to measure circumferential strains. Finally, using the same plane orientations, multislice contrast enhanced MRI (CE-MRI) with an inversion-recovery (IR) sequence covering the whole ventricle was conducted after injection of 0.2 mmol Magnevist^®^ (Bayer, Germany) and waiting for 10 minutes.

SENC-Data were analysed using a dedicated software (Diagnosoft, Paolo Alto, CA, USA). Peak systolic circumferential strain and early diastolic strain rate were measured at each segment in a modified 17 segment model. Early-diastolic strain rate (ECC/s) was defined as the slope over the duration from peak-systole to mid-diastole.

Regional wall motion was evaluated at baseline and at follow-up semi-quantitatively from the SSFP cine sequences by consensus reading of two blinded observers as normokinetic, hypokinetic or akinetic to evaluate functional recovery. CE-MR images were analyzed to quantify the size and transmurality of the scared myocardium using a regular workstation (EWS, Philips, the Netherlands).

## Results

276 segments were analyzed. In 6 segments (2,2%) image quality did not allow adequate data analysis of SENC-images. 157 segments showed normal resting function and 119 segments showed wall motion abnormalities at baseline. 44 segments showed functional recovery at follow, 75 segments did not recover.

Peak systolic circumferential strain in healthy volunteers was -22 ± 3%, early diastolic strain rate was calculated with 122.3 ± 36 E_cc_/s.

**Figure 1:**
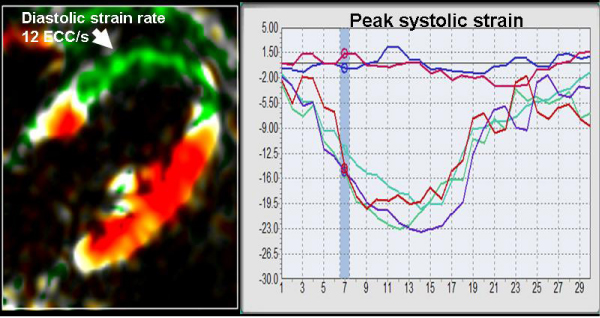
**Colour coded SENC-image of a patient with transmural myocardial infarction with corresponding strain-curve**.

Peak systolic strain values and early diastolic strain rate (figure 1) were significantly different in segments showing functional recovery and in segments without functional recovery (peak systolic strain – 10 ± 1% versus 6 ± 1%, p < 0.01; early diastolic strain rate 75 ± 6 E_cc_/s versus 38 ± 5 E_cc_/s, p < 0.01).

**Figure 2:**
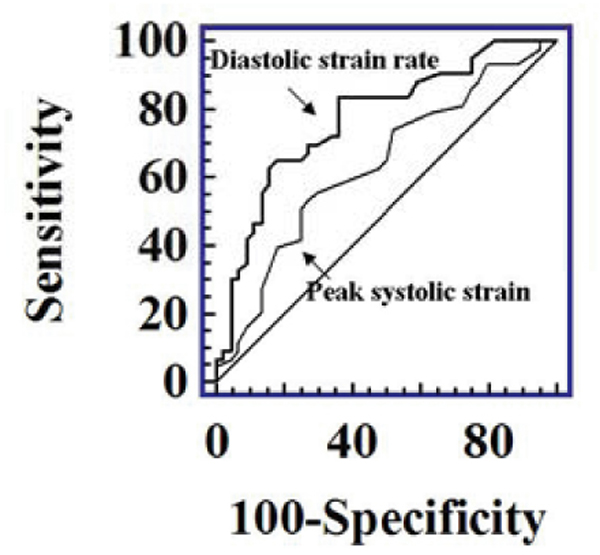
**Receiver-operating characteristic (ROC) curve demonstrates that diastolic strain rate assessed with Strain-Encoded Imaging is more sensitive than peak systolic strain for prediction of functional recovery (diastolic strain rate AUC 0.77 (0.67–0.86); peak systolic strain AUC 0.64 (0.53–0.74); p < 0.05)**.

Diastolic strain rate was more sensitive for prediction of functional recovery than peak systolic strain (figure 2).

Interestingly, peak systolic strain and diastolic strain rate were even more impaired in segments showing microvascular obstruction compared to transmural infarcted segments without microvascular obstruction (p < 0.05 for both).

## Conclusion

SENC allows mechanical characterization of regional myocardial injury. Diastolic function assessed with SENC is more precise in predicting functional recovery after AMI than peak systolic strain.

